# Peptide-Based Materials That Exploit Metal Coordination

**DOI:** 10.3390/ijms24010456

**Published:** 2022-12-27

**Authors:** Giovanni A. Bassan, Silvia Marchesan

**Affiliations:** Chemical and Pharmaceutical Sciences Department, University of Trieste, 34127 Trieste, Italy

**Keywords:** peptides, materials, metals, coordination, complexes, nanostructures, pollutant removal, environmental remediation, sensing, drug delivery

## Abstract

Metal–ion coordination has been widely exploited to control the supramolecular behavior of a variety of building blocks into functional materials. In particular, peptides offer great chemical diversity for metal-binding modes, combined with inherent biocompatibility and biodegradability that make them attractive especially for medicine, sensing, and environmental remediation. The focus of this review is the last 5 years’ progress in this exciting field to conclude with an overview of the future directions that this research area is currently undertaking.

## 1. Introduction

**Figure 1:**
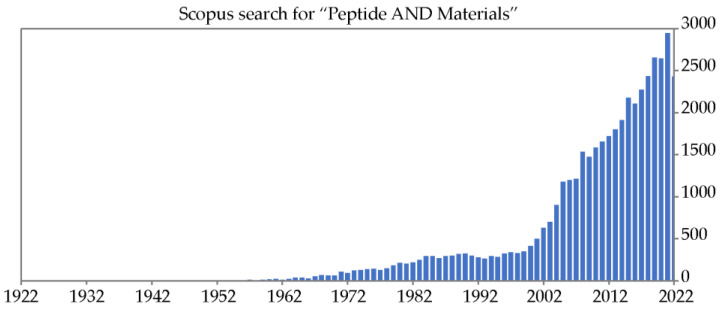
Number of publications in the last century present on Scopus containing “peptide AND materials” in the title, abstract, or keywords. The search was performed on 04-11-22.

**Figure 2:**
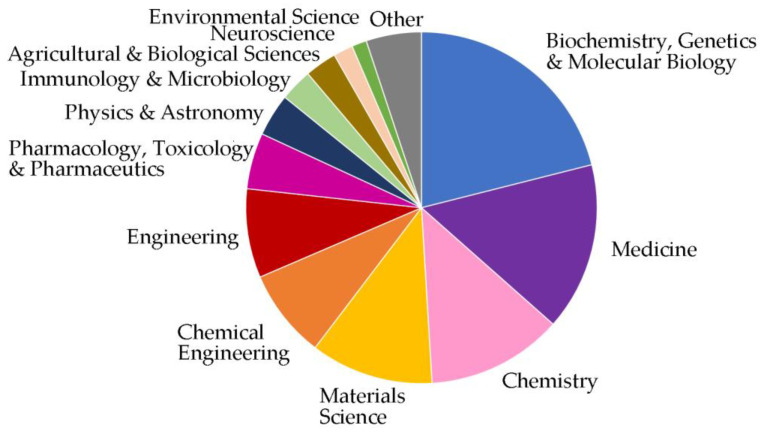
Subject areas of the publications shown in Figure 1.

Peptide-based materials have been attracting researchers’ attention due to their favorable properties, their variety, their ease of preparation also through green methods, and their compatibility with the environment and with living systems. In the last century, over forty-five thousand publications have been produced on the topic, with numbers increasing steadily especially in the last two decades (Figure 1), and it can be surprising to note that the first one dates as back to 1913. However, back then, both knowledge and technology were not mature to understand nanomaterials’ and supramolecular structures. Until the 1970s, the production rate was <100 per year, to then reach >200 in the 1980s, >300 by 1990, and >400 by 2000. Remarkably, by 2010, more than 1.5 k publications were being generated each year, to then almost double to nearly 3 k by 2021. A big impulse to the field was provided by the discovery in 2003 that even a short peptide, as simple as diphenylalanine, was able to form robust nanotubes in mild conditions in water [1]. This finding opened the field also to those with limited or no expertise in organic synthesis and peptide synthesis, and indeed since then the rate of relevant manuscripts per year featured a steep increase. The main research areas have been diverse, spanning from biological sciences and medicine, to chemistry, materials science, engineering, physics, agricultural and environmental sciences (Figure 2) [2].

Peptide-based materials can be classified in different ways, based on the type (i.e., hydrogels, organogels, xerogels, films, adhesives, colloids), on their composition (i.e., purely organic materials, made from peptides alone or in combination with other molecules, or hybrid materials, made from peptides and inorganic components), and, finally, on the leading forces that keep them together. In particular, in this last case, it is possible to distinguish between materials kept together by covalent bonds, and those formed by supramolecular interactions, such as coordination and hydrogen bonds, as well as electrostatic, hydrophobic, and host–guest interactions [3].

**Scheme 1:**
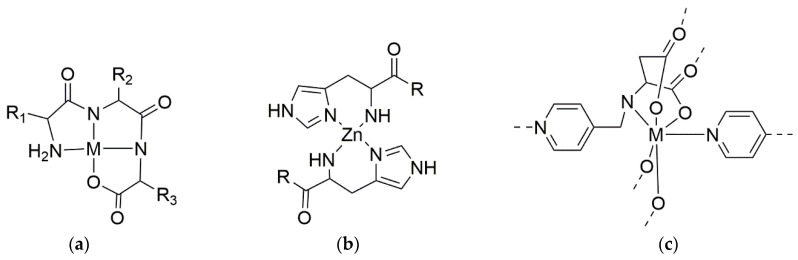
(**a**) Coordination motifs for metal tripeptide complexes, M = Pd, Ni, or Cu [7]. (**b**) Zinc complexes of His-containing peptides [8]. (**c**) Non-natural pyridinyl-amino acid derivative coordinating Co, Zn, or Mn ions [6].

It is well known that peptides can be excellent ligands for a great variety of substances, including metal ions. Different strategies have been developed to chelate these ions with peptides (Scheme 1):

- Amino acids without chelating sidechains, e.g., Phe or Leu, can coordinate metals through their backbone amides and their ammonium and carboxylate termini;
- Those with hydrophilic sidechains containing a Lewis base, e.g., Cys or His, can chelate metals not only through these functional groups, but also through their backbone or termini, so that metal ions act as bridges to enable intermolecular cross-linking;
- Those with chelating ionizable sidechains, e.g., Asp or Lys, can chelate metal ions either through these sidechains, or through their backbone and termini [4];
- Alternatively, the introduction of synthetic heterocycles, such as pyridine units, enabled the formation of complexes (e.g., with palladium ions) to hold together complex supramolecular architectures [5,6].

**Figure 3:**
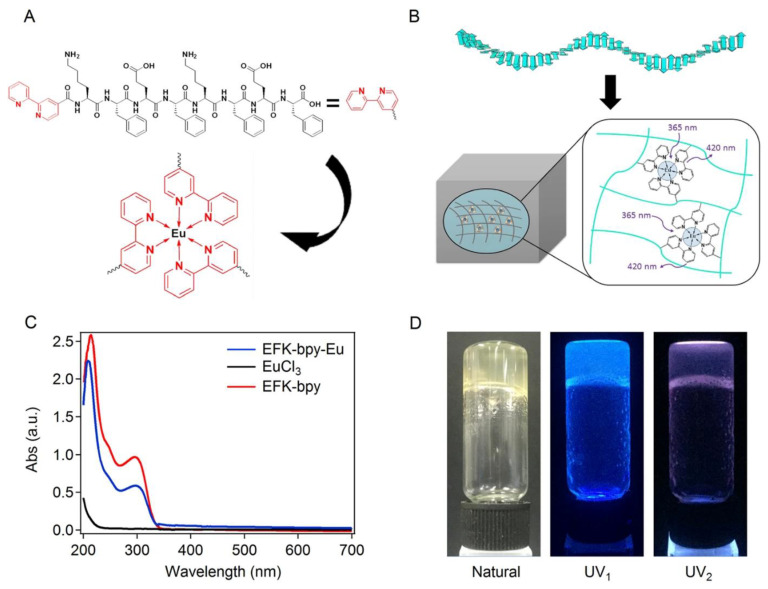
Coordination with Europium yields cross-linked luminescent hydrogels from a peptide with bipyridinyl moieties. (**A**) peptide structure with metal-binding bipyridinyl group in red. (**B**) schematic of peptide self-assembly into stacks and cross-linking with the metal. (**C**) UV-visible absorbance spectra of the cross-linked peptide gel (EFK-bpy-Eu), and the metal (EuCl_3_) or peptide (EAK-bpy) components alone. (**D**) photographs of the luminescent gels under natural and UV light. Reproduced from [9] under a Creative Commons license.

These features, coupled with the typical dynamism and reversibility of coordination bonds, as well as their high directionality and defined geometry, open the way to new avenues towards materials with emerging behavior from the metal–ion coordination with peptides. Firstly, the formation of metal–peptide complexes could be a trigger for the sol-to-gel transition (Figure 3), or the other way around. As an example, Figure 3 shows the inclusion of a bis-pyridinyl moiety to a peptide forming β-sheets as a strategy to cross-link peptide stacks into a luminescent hydrogel in the presence of Eu (III) [9]. Secondly, it could introduce the dependence of this transition from different chemical or physical stimuli such as pH, temperature, ionic strength, light, concentration, or changes in the composition of the environment [10]. Thirdly, the coordination with metals can be used to tune and refine the characteristics of a given peptide-material, such as stress resistance, stiffness, self-healing capability, redox, or catalytic activity. All these advantageous features contribute to make metal coordination a very interesting and useful approach to explore new frontiers in the production of innovative, low-toxicity materials for a wide range of applications. These include, but are not limited to, 3D printing, controlled-release agents for bioactive compounds, imaging, environmental remediation, catalysis, and sensing [11].

**Figure 4:**
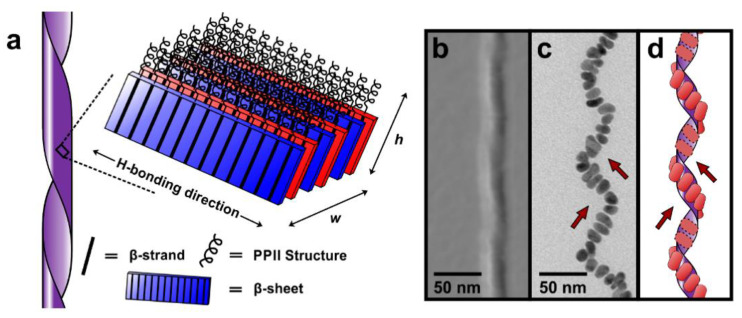
(**a**) Cartoon showing peptide assembly into a nanoribbon. (**b**) AFM image of the resulting assembled fiber. (**c**) TEM image of the gold nanorods oriented (arrows) into a helical pattern. (**d**) Proposed model of the gold nanorods associated to the outer surface of the peptide ribbon. Adapted with permission from [13]. Copyright © 2016, American Chemical Society.

Furthermore, coordination could prove useful to template metal nanoparticle (NP) [12], nanorods [13], or nanocluster [14] formation in situ through a green process, eventually to generate a hybrid material for advanced applications [15,16]. As an example, Figure 4 shows a cartoon for peptide self-assembly into nanoribbons, and microscopy images that revealed how they were used to template the in situ reduction of gold into oriented nanorods, yielding a chiroptical material [13]. Alternatively, interesting topological features could be introduced to attain soft or hard matter with selective and dual porosities, for instance through the generation of cages or pores with selective binding abilities for molecular guests, beyond the mesh between peptide chains [17,18,19,20].

In this review, we will focus on the recent developments of the last five years on peptide materials that feature metal coordination based on their possible applications, especially in medicine, but also for environmental remediation and sensing. Readers are referred to reviews dating 2017–2018 for coverage of earlier findings in these fields [21,22]. In this work, we will not cover applications in catalysis since extensive reviews have just been published on this topic [23,24,25,26,27].

## 2. Metal–Peptide Materials in Medicine

**Figure 5:**
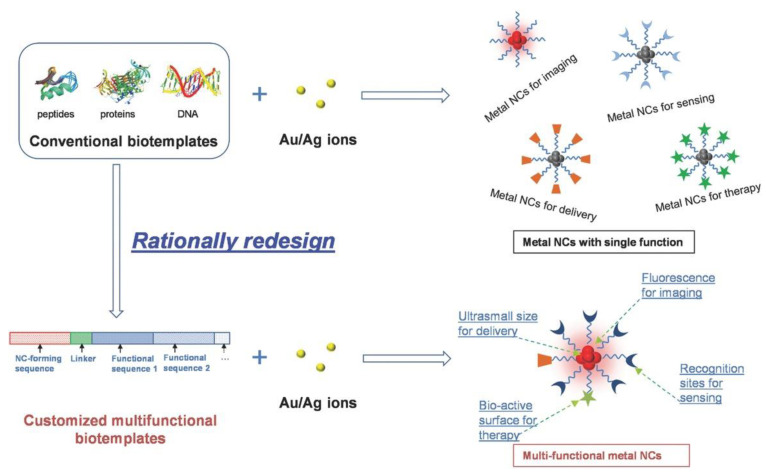
Peptides and metal nanoclusters (NCs) find various applications in medicine, including imaging, sensing, delivery, and therapy. Reproduced with permission from [32], © 2016 WILEY-VCH Verlag GmbH & Co. KGaA, Weinheim.

Peptide-based materials have been a hot topic in the search for innovative techniques for diagnosing and treating patients, that could be safer, easier to implement, and less invasive relative to traditional options, thanks to their similarity to natural biomolecules and their assembled structures [28,29,30]. Thanks to rational design, they found various applications in medicine (Figure 5) [31,32], and a special focus has been placed on the controlled delivery of therapeutics [33,34,35,36], cancer therapy [37,38], wound healing [39], tissue engineering [39], imaging [40,41] and theranostics [42], treatment of infections [43], sensing [44,45], and vaccine development [46,47]. Moreover, the interaction between the peptide and the metal of interest could occur both before the contact of the material with cells, or when the peptide reaches the target area. In this latter case, metal coordination could be triggered by local physiological or pathological conditions (e.g., pH, ion concentration), so as to change the material behavior and even its structure, in terms of aggregation state and porosity [48].

### 2.1. Minimalistic Systems Based on Dipeptides Interacting with Metal Ions for Drug Delivery

**Figure 6:**
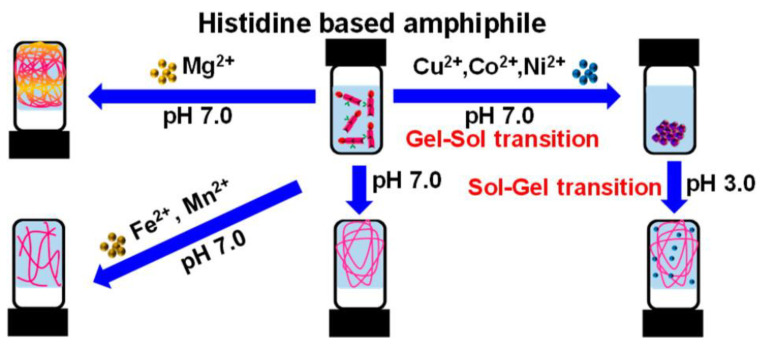
Metal-cation responsive behavior of supramolecular hydrogels composed of Fmoc-dipeptides with His. Reproduced with permission from [51]. Copyright © 2019, American Chemical Society.

Since the report by Reches and Gazit on the self-assembly of diphenylalanine into nanotubes and its potential use as templates to cast silver nanowires [1], there has been a great interest in minimalistic dipeptide systems and their use in combination with metals. In particular, a popular approach to attain self-assembly of simple amino acids or dipeptides is based on the inclusion of aromatic N-caps [49]. Among these, the fluorenylmethyloxycarbonyl (Fmoc) moiety has been by far the most popular, also because Fmoc-amino acids are commercially available, being the building blocks for solid-phase peptide synthesis [50]. In a recent progress, Fmoc-dipeptides that could self-assemble into metal–ion responsive hydrogels (Figure 6) were studied for possible applications as drug carriers. Sharma et al. [51] reported the case of Fmoc-His-Phe, Fmoc-His-Leu, and Fmoc-His-Val that formed hydrogels when the pH was slowly decreased from alkaline to neutral values. These hydrogels demonstrated to be responsive to the presence of different salts of divalent metal ions, i.e., Ni^2+^, Co^2+^, Cu^2+^, Fe^2+^, and Mn^2+^. The presence of these metals modulated the properties of the hydrogels in various ways. In the case of Ni^2+^, Co^2+^, and Cu^2+^, the interaction caused the gel-to-sol transition, with a new sol-to-gel transition with pH lowering to 3. In the case of Fe^2+^ and Mn^2+^, presence of the ions weakened the gels, although the strength of their interaction with the peptides was not sufficient to induce a transition to sol. These behaviors could enable the controllable release of bioactive compounds loaded in the gels in areas with a higher concentration of these ions.

In the case of Fmoc-Phe-Phe, metal binding could exert different effects depending on the metal ion type (i.e., Na^+^, K^+^, Zn^2+^, Cu^2+^, Fe^3+^, Al^3+^), and concentration relative to the dipeptide. The coordination affected the biomolecular secondary structure towards β-sheets, random coils, or superhelices, and, consequently, gelation ability, kinetics, and rheology [52]. 

### 2.2. Longer Peptides Interacting with Metal Ions for Drug Delivery

Elongation of the peptide sequence by adding further amino acids is often convenient to enable the formation of secondary structures, or to introduce protein-inspired bioactive motifs and fine-tune the biological properties of a given material. Peptide-based materials and, in particular, supramolecular hydrogels, have been widely studied as delivery agents of therapeutics [53]. Their interactions with metal ions offer an attractive strategy to trigger their cargo release in situ [21]. Ca^2+^ or Mg^2+^ proved effective to enhance the rheological properties of a collagen-inspired peptide anion Nap-Phe-Phe-Gly-Asp-Hyp, inducing its gelation at physiological pH. The material showed better cytocompatibility relative to the peptide alone, with increased cell viability and agglomeration [54]. A similar interesting behavior was observed using Ca^2+^ as a trigger for gelation with three peptide amphiphiles containing disulfide bridges between poly-L-Cys and 3-mercaptopropionic acid. The pendant carboxylate groups were used to bind cisplatin for its sustained release, which could be enabled using either pH changes or redox triggers. These peptide amphiphiles could form micelles or nanofibrils, depending on their concentration and pH value. Both structures could interact with Ca^2+^ to adopt a gelling β-sheet structure. Furthermore, the material displayed good cytocompatibility in vitro [55].

Micelles could be obtained from the union of different peptides and peptoids based on the sequence poly(Sar)-*block*-poly(Glu), where Sar indicates sarcosine. In solution, these copolymers self-assembled in micelles that could be cross-linked with *cis*-diaquabis(2,2′-bipyridine)-ruthenium(II) complex ([Ru(bipy)_2_(H_2_O)_2_]^2+^) or with *cis*-diaquabis(2,2′-biquinoline)-ruthenium(II) complex ([Ru(biq)_2_(H2O)_2_]^2+^). The micelle morphology was tunable from spherical to more elongated, depending on the nature of the copolymer and the complex used. Importantly, the cross-linked systems demonstrated photocleavable behavior: blue light-irradiation resulted in the complete release of Ru(II) from the micelles with the first complex. These systems were tested on skin tumoral cells HuH-7 and demonstrated a higher absorption for Ru(II) complexes, and an enhanced cytotoxicity that could enable their use as photo-controlled carriers for cancer therapy [56].

Cisplatin could also be used to induce peptide gelation. Wu et al. reported that NapPhe-Phe-Tyr-Glu-Arg-Gly-Asp could form a hybrid hydrogel trough both coordination bonds between Asp, Glu and Arg sidechains with Pt^2+^ ions of cisplatin, and ionic interactions with NPs of the hybrid alginate–irinotecan. This material performed well in terms of injectability, loading and controlled release of the two drugs separately, depending on the concentration of alginate present in the local environment [57].

A different use of Ca^2+^ was envisaged in the case of the decapeptide Nap-Gly-Phe-Phe-Tyr-Gly-Arg-Gly-Asp-His-His that spontaneously self-assembled into a supramolecular gel with good biocompatibility and wide possibilities for further functionalization. One limitation of this material was its low stability for long periods of time, that was addressed by using Ca^2+^ for cross-linking with other gelators, such as alginate. This strategy enhanced the biocompatibility of the system, and it introduced also the possibility to obtain reversible transitions to enable its injectability and to increase cell adhesion [58].

Ca^2+^ induced the sol-to-gel transition of the peptide Fmoc-Phe-Phe-pSer^C^-(oNB)-PEG that contained *ortho*-nitrobenzyl (oNB) protected phosphonated Ser. Gelation could be triggered also by Zn (II), Co (II) and Cu (II) ions, but only the gel obtained with Ca^2+^ showed new features in terms of photo-activity. This hydrogel was responsive to the concentration of the metal and light irradiation at 365 nm disrupted the gel, which reformed when left in the dark. The authors then applied the material for the loading and release of the anti-tumoral drug doxorubicin [59].

### 2.3. Peptides and Metal Ions for Tissue Regeneration

Peptides have attracted great interest for their ability to self-assemble into nanofibrillar hydrogels that mimic the extracellular matrix, thus providing an ideal scaffold for cells’ growth and tissue regeneration [60]. In particular, the use of metal coordination offers a convenient strategy to improve the mechanical properties of the hydrogels and provide them with self-healing behavior [61,62]. Patel et al. developed a thermogel based on a poly-Ala oligopeptide functionalized with PEG that could coordinate Fe(III) ions with a crown-ether like system. Gelation could be obtained at 37 °C, at physiological pH, and presence of the metal ions enhanced the material properties. When tested on neuronal staminal cells, the gel proved to be cytocompatible, it promoted cell aggregation, with consequent slow release of metal ions (i.e., 10% of the total iron ions was observed after 21 days for different Fe(III) concentrations). Interestingly, cells displayed an increased release of different differentiation factors induced by the iron-containing gel. Overall, this material was thus envisaged for applications as an injectable agent to promote neuronal-cell differentiation for regenerative medicine [63].

### 2.4. Peptides and Metal Ions for Wound Healing

**Figure 7:**
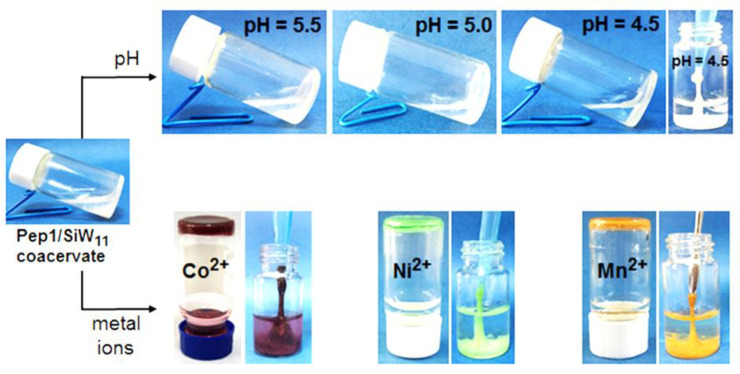
pH and metal–ion responsiveness of a coacervate based on a peptide (Pep1) and POMs (SiW_11_). Adapted with permission from [66]. Copyright © 2019, American Chemical Society.

For wound healing applications, silver is a traditional metal that has been added to peptide hydrogels for its antibacterial properties [64]. Recently, more complex systems can be built using complex tridimensional metal structures: polyoxometalates (POMs), a class of polynuclear metal-oxo anions of early-transition metal ions in high-oxidation states, e.g., Mo(VI) or W(VI) [65]. These polyanions could be combined with polycationic peptide gelators towards denser fibrillar networks that turned the gels into adhesives. In particular, the tripeptides Gly-His-Lys, Gly-Phe-Lys, and Gly-Val-Lys were combined with POMs to yield underwater coacervates in solution. By adding divalent ions, such as Mn^2+^, Ni^2+^, or Co^2+^, it was possible to induce the aggregation of the coacervates into strong underwater adhesives (Figure 7) that could be envisaged for applications in wound dressings [66].

### 2.5. Peptides and Metal Ions for Antimicrobial Materials

Antimicrobial peptides have been inspiring research activities towards bioactive coatings and adhesives for biomedical use [67], also beyond wound healing applications [68]. Minimalistic systems such as amino-acid derivatives [69,70] or dipeptides [71,72,73,74] can be antimicrobial too, although their activity can be sometimes too mild for practical applications [75]. Therefore, a combination of bioactive peptides with metal ions and nanostructures for additive or synergistic effects to fight infections can be a convenient strategy [76,77,78]. In this regard, copper, silver, and zinc clearly play an elected role for their established antimicrobial activity and possibility of production with reasonable, or even low, costs [79]. In particular silver, also in its NP form, is the most widely studied [80].

The simplest materials that have been studied for biomedical application are hydrogels made of a single amino acid capable of self-assembly into a supramolecular stable structure, driven by an external stimulus that could be its concentration, the pH, or the ionic strength of the solution. These gels were highly biocompatible, thanks to the nature of their constituents, and they could be very versatile in functionalization and use. It has been reported that four Fmoc-amino acids (i.e., Fmoc-Pro, Fmoc-His, Fmoc-Ala, and Fmoc-Leu) could form hydrogels only when coordinated with Ag^+^, also promoting the formation of ultrasmall silver NPs, as demonstrated by XPS and TEM studies [81]. These materials showed great potential for the loading and release of small antimicrobial drugs under controlled conditions, and they demonstrated themselves antimicrobial activity, thanks to the presence of Ag^+^ ions and NPs that could interact with the hydrophobic bacterial membrane, inducing cytoplasmatic leaking outside the cell.

D’Souza et al. designed a peptide sequence with two units of 3′-pyridyl-Ala (3′PyA) to coordinate Ag^+^ and gel. Ag(I) tendency to form two-coordinate linear complexes was exploited to crosslink the nonapeptide (3′-PyA)-Leu-Arg-Leu-Arg-Leu-Arg-Leu-(3′-PyA) into a gel with a 15-fold increase in storage modulus, relative to the peptide alone without silver. The gel demonstrated a higher antimicrobial activity in the presence of Ag(I) both against Gram-positive (*S. auerus*) and Gram-negative (*E. coli*) bacteria, with good cytocompatibility on mammalian cells in vitro [82]. Shnaider et al. reported that also nanofibrillar silk-hydrogel composites with silver NPs exerted good antimicrobial activity and biocompatibility. In particular, the materials were produced with a microfluidic process that ensured high reproducibility and homogeneity, and they exerted a two-step activity, first by promoting bacterial adhesion, and then their eradication, in vitro and in vivo [83]. Iudin et al. described core-shell NPs that featured a silver core, and a poly(Glu) shell capable of binding the antimicrobial peptide polymyxin. Interestingly, a synergistic effect was noted between the components, as demonstrated by a detailed analysis of polymyxin release and minimal bioactive concentration [84]. Besides silver, also nanostructured copper was recently combined with bioactive peptides. In particular, copper nanoclusters were capped with the antioxidant tripeptide glutathione for increased stability in aqueous solutions, and they exhibited antibacterial activity on *E. coli* [85].

### 2.6. Amyloid Beta (Aβ) Fibrillation Inhibitors Based on Metal–Ion–Peptide Nanostructures

**Figure 8:**
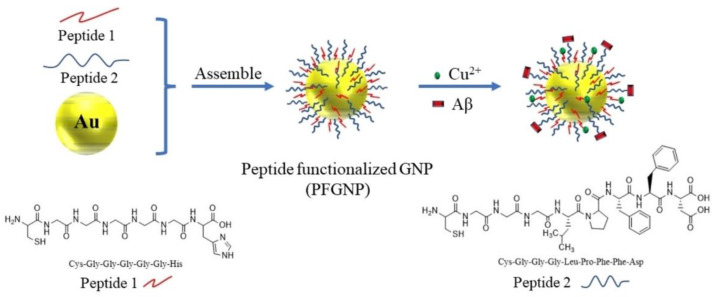
Metal-chelating peptides were used to coat gold NPs and bind Cu^2+^ ions to inhibit Aβ fibrillation. Reproduced with permission from [114]. Copyright © 2022 Wiley-VHCA AG, Zurich, Switzerland.

Peptide-based nanomaterials have been envisaged also as inhibitors of pathological amyloid fibrillation [86,87,88]. Often, a sequence is designed to contain the Phe-Phe motif for recognition of, and binding to, Aβ as well as an amino acids that can act as β-breaker to disrupt the formation of cross-β structures that are typical of amyloids. In the past, for instance, this approach proved useful with proline, as described by Soto et al. [89]. More recently, a simple tripeptide L-Pro-D-Phe-L-Phe formed nanoparticles and could inhibit fibrillation in vitro, whilst displaying good cytocompatibility on mammalian cells, and high resistance against protease-mediated hydrolysis [90]. Furthermore, there is emerging evidence that biometal (especially Fe, Cu, and Zn) dyshomeostasis and metal-amyloid interactions lead to the pathogenesis of Alzheimer’s disease, thus metal-chelation strategies have also been used to inhibit Aβ fibrillation [91,92,93,94]. Co (II) [95,96], Pt (II) [97,98,99], Ru (II) [100,101], and heterometallic Pt(II)-Ru(II) [102] complexes proved effective too for this purpose, also through chelation of Cu (II) that was captured from Aβ [103]. It is thus not surprising that peptides binding Cu(II) [104,105,106,107,108,109,110], Zn(II) [111,112,113], and Cd(II) [112] have been successfully designed to inhibit Aβ fibrillation. Researchers have sought to combine gold NPs with Cu (II) chelating peptides to develop effective inhibitors (Figure 8) [114]. In this case, the peptides served three functions: (1) as capping agents to stabilize the gold NPs, (2) as bait to bind the Aβ peptide through the Phe-Phe motif and inhibit fibrillation using the sequence Leu-Pro-Phe-Phe-Asp, as well as (3) capture Cu(II) from amyloid plaques using the Gly-Gly-His motif.

## 3. Metal–Peptide Materials for Environmental Remediation

Recently, the interest in developing new methods for removing pollutants from different environments has grown strong, especially for the treatment of waste waters of industrial processes. The discovery that simple amino acids and peptides could gel oils from mixtures with water raised in interest in their use to remediate polluted environments from oil spills [115]. In this field, peptide hydrogels found wide applicability to remove dangerous metal ions and organic molecules too, such as ionic dyes, in a safe, fast, and selective way, often giving, in addition, the opportunity to recycle the material different times before it expires. Systems as simple as dipeptides have been designed for this purpose. For instance, the protected dipeptide myristil-Trp-Phe could form both hydrogels and organogels in different solvents, including petroleum, kerosene, diesel, and petroleum ether. The organogels demonstrated a great absorption ability of toxic organic molecules, while the hydrogels could remove up to 98.8% of toxic heavy metal ions, such as Pb^2+^ and Cd^2+^. Extraction with ethyl acetate enabled recovery of the gelator for its application in another two cycles of absorption. Solvent removal yielded a stable xerogel that could perform great absorption also towards aqueous solutions of common organic ionic dyes [116].

A pyrene–peptide amphiphilic conjugate was designed with two Glu residues for metal coordination through their sidechains, while the polyaromatic unit enabled self-assembly and gelation. Several metal ions were tested for their effects on the gelation ability of the amphiphile, with Zn^2+^ yielding the best soft material for the selective absorption and removal of cationic dyes (i.e., methylene blue), as opposed to anionic dyes (i.e, methyl orange), present individually or in mixtures [117].

A more complex system was proposed by Huang et al. to remove only Cd(II) ions in a selective way from waters. This system was based on a hybrid PNIPAM-CadRP, obtained from the union of the Cd-binding peptide CadRP (which was a fragment of the protein that regulates its concentration CadR), with poly(*N*-isopropyl)acrylamide (PNIPAM). At temperatures below 34 °C, this peptide with Cd(II) ions formed a well-swollen material that changed its porosity when the temperature was raised. As a result, the accessibility to the internal peptide and the release of the ions was also different. This phenomenon rendered the system suitable for treating waters containing cadmium ions, also thanks to its reusability for various cycles [118].

## 4. Metal–Peptide Materials for Sensing

**Figure 9:**
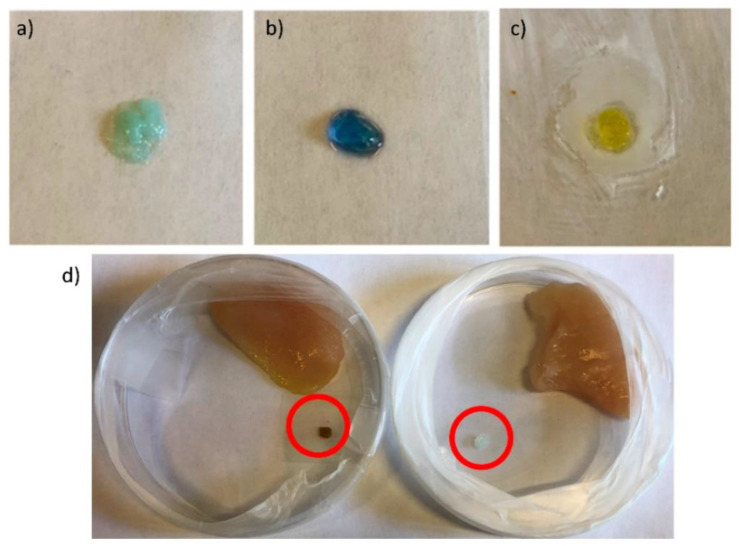
Application of peptide-Cu(II) gel for the monitoring of food freshness. Images of pyrene-peptide-Cu^2+^ gel (**a**) as prepared, (**b**) after exposure to ammonia vapors, and (**c**) after exposure to HCl vapors. (**d**) Colorimetric responses of the gel (red circles) when exposed to chicken meat stored at 25 °C (left) and 4 °C (right). Reproduced from [117].

The combination of peptides with metals has been studied for applications in sensing too. In the past, peptides attracted great interest as biorecognition motifs that could be coupled to electrochemical transducers for various sensing applications, as recently reviewed [119]. More recently, self-assembling peptides forming ordered arrays, films, and nanotubes, have been coupled also to carbon nanostructures to create innovative sensing platforms that could exploit also other properties of the peptides, besides biorecognition [120]. Peptides and metals have been combined in various ways. For instance, peptide–metal conjugates have been applied to biosensing [121]. Peptides have been used for the sensing of metals too [122]. Mba’s research group has recently reported an interesting application of a pyrene–peptide conjugate gel that coordinated Cu(II) ions through Glu sidechains, for monitoring food freshness [117]. The colorimetric response of copper to changes in the coordination environment was thus exploited to sense amines, since their production is a useful indicator of the spoilage of meat products [123]. Indeed, the hydrogel manifested different colors depending on the type of amine present, and turned from light blue to dark brown when exposed to spoiled chicken breast (Figure 9).

Another pyrene–peptide conjugate was recently reported for sensing, this time for Ca^2+^ ions. The sequence Val-Pro-Gly-Lys-Gly was used in this work to obtain a thermo-responsive self-assembled system that formed spherical aggregates in water, that gelled in response to octafluoronaphthalene, thanks to the establishment of aromatic interaction between the electron-rich and the electron-poor aromatic units. This gel was responsive to the presence of Ca^2+^ through coordination and changes in its critical gelation concentration. For these reasons, this system was envisaged as a sensor for the presence of calcium ions [124]. Wang et al. reported in this field the case of a xerogel derived from the union of the peptide (D-Asp)_5_ with silver nanoclusters [Ag_9_(mba)_9_] (mba = 2-mercaptobenzoate). The coordination of the peptides with Ag^+^ ions of the clusters led to a luminescent material, and the large Stokes shift (≈140 nm) and a fluorescence time in the scale of microseconds (6.1 µs) suggested that the hydrogel was a phosphor. This material was able to selectively recognize small biological molecules, in particular L-Arg and D-Arg that could totally quench the phosphorescence of the gel. Studies on the effect of temperature demonstrated that this system was stable up to 200 °C, making it a very useful sensor for these amino acids [125].

## 5. Conclusions

In this overview, we showed the latest developments in the production of new materials derived from the coordination of metal ions to different peptides in order to obtain new materials for applications in medicine, pollutants’ removal, and sensing (Table 1). From the summary shown in Table 1, it is evident that hydrogels are by far the most popular type of matter under study, and that metal ions in the divalent state are those mostly studied to cross-link the peptides. There is thus still a clear gap pertaining in particular transition-state metals, with studies on ruthenium and platinum being very recent additions to the field.

As discussed in this review, these new materials are gaining more interest for their features, especially in the field of biomedicine, since they provide great opportunities for the development of safe and biocompatible drug carriers, with the excellent possibility to control the release of the molecules that they are carrying. Moreover, some of these materials are also useful for the development of functional biofilms containing different types of cells, or new hemostatic agents suitable for wound healing. The combination of responsiveness to local-environment conditions (e.g., pH, metal ions, etc.) with optical and/or electronic properties conferred by the metal ions (e.g., photo-activity, luminescence), place these materials into an ideal spot to develop theranostics, where advanced drug delivery is combined with diagnostics, to address unmet challenges in medicine, such as those posed by aggressive cancer types.

Besides applications in medicine, these materials show interesting behavior also in the treatment of waste waters, since the peptides can form gels from coordination with toxic ions, thus providing convenient tools for their removal, together with organic dyes, since the formed peptide–metal gels can trap these molecules inside them. Clearly, the scale-up and industrial implementation of this type of soft matter for environmental remediation still faces practical challenges to solve, such as robustness of the hydrogels to undergo harsh industrial conditions, and multiple cycles of use, as well as the development of convenient workflows for large-scale applications. Sensing is another area that benefits from the optoelectronic and robustness properties of metals with the biorecognition ability of peptides. However, also in this case, key challenges to be addressed pertain the robustness and reliability of biosensors through multiple cycles under conditions pertinent to commercial use, and reproducibility of biosensor production and performance to meet industrial needs. Nevertheless, this field is moving at a fast pace also towards exciting new areas such as bioelectronics, and innovative ways to store, convert, and transfer energy in a green manner [126,127,128,129]. Finally, the combination of minimalistic peptides or amino acids with metal ions to provide porous materials and metal-organic frameworks [130,131,132] is attracting a lot of attention and could provide green alternatives for the future entrapment of gases or other species, as well as for entioselective separation, although this field is still in its infancy.

## Figures and Tables

**Table 1:** Summary of metal–peptide-based materials discussed in this review.

Metal Ion	Peptide	Material	Application	Ref.
Na^+^	Fmoc-Phe-Phe	Hydrogel	DNA biochip	[52]
K^+^	Fmoc-Phe-Phe	Hydrogel	Medicine	[52]
Ag^+^	Fmoc-Pro	Hydrogel	AM ^1^/Drug delivery	[81]
	Fmoc-His	Hydrogel	AM ^1^/Drug delivery	[81]
	Fmoc-Ala	Hydrogel	AM ^1^/Drug delivery	[81]
	Fmoc-Leu	Hydrogel	AM ^1^/Drug delivery	[81]
	(3′-PyA)-Leu-Arg-Leu-Arg-Leu-Arg-Leu-(3′-PyA)	Hydrogel	AM ^1^	[82]
	Silk fibroin	Nanocomposite	AM ^1^	[83]
	Poly(Glu)/polymyxin	Nanoparticles	AM ^1^	[84]
	(D-Asp)_5_	Hydrogel	Arg sensing	[125]
Mg^2+^	Nap-Phe-Phe-Gly-Asp-Hyp	Hydrogel	Drug delivery	[54]
Ca^2+^	Nap-Phe-Phe-Gly-Asp-Hyp	Hydrogel	Drug delivery	[54]
	Poly-Cys amphiphile	Hydrogel	Drug delivery	[55]
	Nap-Gly-Phe-Phe-Tyr-Gly-Arg-Gly-Asp-His-His	Hydrogel	Drug delivery	[58]
	Fmoc-Phe-Phe-pSer^C^-(oNB)-PEG	Hydrogel	Drug delivery	[59]
	Pyrenyl-Val-Pro-Gly-Lys-Gly	Hydrogel	Ca^++^ sensing	[124]
Mn^2+^	Fmoc-His-Phe	Hydrogel	Drug delivery	[51]
	Fmoc-His-Leu	Hydrogel	Drug delivery	[51]
	Fmoc-His-Val	Hydrogel	Drug delivery	[51]
	Gly-His-Lys	Adhesive	Wound healing	[66]
	Gly-Phe-Lys	Adhesive	Wound healing	[66]
	Gly-Val-Lys	Adhesive	Wound healing	[66]
Fe^2+^	Fmoc-His-Phe	Hydrogel	Drug delivery	[51]
	Fmoc-His-Leu	Hydrogel	Drug delivery	[51]
	Fmoc-His-Val	Hydrogel	Drug delivery	[51]
Co^2+^	Fmoc-His-Phe	Hydrogel	Drug delivery	[51]
	Fmoc-His-Leu	Hydrogel	Drug delivery	[51]
	Fmoc-His-Val	Hydrogel	Drug delivery	[51]
	Gly-His-Lys	Adhesive	Wound healing	[66]
	Gly-Phe-Lys	Adhesive	Wound healing	[66]
	Gly-Val-Lys	Adhesive	Wound healing	[66]
	Fmoc-Phe-Phe-pSer^C^-(oNB)-PEG	Hydrogel	Drug delivery	[59]
Ni^2+^	Fmoc-His-Phe	Hydrogel	Drug delivery	[51]
	Fmoc-His-Leu	Hydrogel	Drug delivery	[51]
	Fmoc-His-Val	Hydrogel	Drug delivery	[51]
	Gly-His-Lys	Adhesive	Wound healing	[66]
	Gly-Phe-Lys	Adhesive	Wound healing	[66]
	Gly-Val-Lys	Adhesive	Wound healing	[66]
Cu^2+^	Fmoc-His-Phe	Hydrogel	Drug delivery	[51]
	Fmoc-His-Leu	Hydrogel	Drug delivery	[51]
	Fmoc-His-Val	Hydrogel	Drug delivery	[51]
	Fmoc-Phe-Phe	Hydrogel	Drug delivery	[52]
	Fmoc-Phe-Phe-pSer^C^-(oNB)-PEG	Hydrogel	Drug delivery	[59]
	Glutathione	Nanoclusters	AM ^1^	[85]
	Cys-(Gly)_5_-His, Cys-(Gly)_3_-Leu-Pro-Phe-Phe-Asp	Nanoparticles	Amyloid inhibition	[114]
	Phe-Glu-Phe-Glu-Gly-pyrene	Hydrogel	Amine sensing	[117]
Zn^2+^	Fmoc-Phe-Phe	Hydrogel	DNA biochip	[52]
	Fmoc-Phe-Phe-pSer^C^-(oNB)-PEG	Hydrogel	Drug delivery	[59]
	Phe-Glu-Phe-Glu-Gly-pyrene	Hydrogel	Pollutant capture	[117]
Ru^2+^	Poly(Sar)-*block*-poly(Glu)	Micelles	Drug delivery	[56]
Cd^2+^	Myristil-Trp-Phe	Hydrogel	Pollutant capture	[116]
	PNIPAM-CadRP	Hydrogel	Pollutant capture	[118]
Pt^2+^	Poly-Cys amphiphile	Hydrogel	Drug delivery	[55]
	Nap-Phe-Phe-Tyr-Glu-Arg-Gly-Asp	Hydrogel	Drug delivery	[57]
Pb^2+^	Myristil-Trp-Phe	Hydrogel	Pollutant capture	[116]
Al^3+^	Fmoc-Phe-Phe	Nanofibrils/spheres	Medicine	[52]
Fe^3+^	Fmoc-Phe-Phe	Nanofibrils/spheres	Medicine	[52]
	PEG-poly-Ala	Hydrogel	Tissue regeneration	[63]
Mo^4+^	Gly-His-Lys	Coacervate	Wound healing	[66]
	Gly-Phe-Lys	Coacervate	Wound healing	[66]
	Gly-Val-Lys	Coacervate	Wound healing	[66]
W^4+^	Gly-His-Lys	Coacervate	Wound healing	[66]
	Gly-Phe-Lys	Coacervate	Wound healing	[66]
	Gly-Val-Lys	Coacervate	Wound healing	[66]

^1^ AM = antimicrobial.

## Data Availability

Not applicable.

## References

[1] Reches M., Gazit E. (2003). Casting metal nanowires within discrete self-assembled peptide nanotubes. Science.

[B2-ijms-24-00456] 2.Scopus search on 04-11-22 for “Peptide AND Materials” on title, abstract, or keywords.

[3] Maldonado N., Amo-Ochoa P. (2021). Advances and novel perspectives on colloids, hydrogels, and aerogels based on coordination bonds with biological interest ligands. Nanomaterials.

[4] Sóvágó I., Kállay C., Várnagy K. (2012). Peptides as complexing agents: Factors influencing the structure and thermodynamic stability of peptide complexes. Coord. Chem. Rev..

[5] Zou R., Wang Q., Wu J., Wu J., Schmuck C., Tian H. (2015). Peptide self-assembly triggered by metal ions. Chem. Soc. Rev..

[6] Wang C., Zhang N., Hou C.-Y., Han X.-X., Liu C.-H., Xing Y.-H., Bai F.-Y., Sun L.-X. (2020). Transition metal complexes constructed by pyridine–amino acid: Fluorescence sensing and catalytic properties. Transit. Met. Chem..

[7] Monger L.J., Razinkov D., Bjornsson R., Suman S.G. (2021). Synthesis, characterization, and reaction studies of Pd(II) tripeptide complexes. Molecules.

[8] Förster M., Vahrenkamp H. (1995). Zinc complexes of histidine-containing di- and tripeptides. Chem. Ber..

[9] Xia Y., Xue B., Qin M., Cao Y., Li Y., Wang W. (2017). Printable fluorescent hydrogels based on self-assembling peptides. Sci. Rep..

[10] Shao T., Falcone N., Kraatz H.-B. (2020). Supramolecular peptide gels: Influencing properties by metal ion coordination and their wide-ranging applications. ACS Omega.

[11] Khare E., Holten-Andersen N., Buehler M.J. (2021). Transition-metal coordinate bonds for bioinspired macromolecules with tunable mechanical properties. Nat. Rev. Mater..

[12] Bellotto O., Cringoli M.C., Perathoner S., Fornasiero P., Marchesan S. (2021). Peptide gelators to template inorganic nanoparticle formation. Gels.

[13] Merg A.D., Boatz J.C., Mandal A., Zhao G., Mokashi-Punekar S., Liu C., Wang X., Zhang P., van der Wel P.C.A., Rosi N.L. (2016). Peptide-directed assembly of single-helical gold nanoparticle superstructures exhibiting intense chiroptical activity. J. Am. Chem. Soc..

[14] Qiao Z., Zhang J., Hai X., Yan Y., Song W., Bi S. (2021). Recent advances in templated synthesis of metal nanoclusters and their applications in biosensing, bioimaging and theranostics. Biosens. Bioelectron..

[15] Rozhin P., Melchionna M., Fornasiero P., Marchesan S. (2021). Nanostructured ceria: Biomolecular templates and (bio)applications. Nanomaterials.

[16] Saif B., Yang P. (2021). Metal–protein hybrid materials with desired functions and potential applications. ACS Appl. Bio Mater..

[17] Jeong S., Lee K., Yoo S.H., Lee H.-S., Kwon S. (2022). Crystalline metal-peptide networks: Structures, applications, and future outlook. ChemBioChem.

[18] Jahović I., Zou Y.-Q., Adorinni S., Nitschke J.R., Marchesan S. (2021). Cages meet gels: Smart materials with dual porosity. Matter.

[19] Dey S., Misra R., Saseendran A., Pahan S., Gopi H.N. (2021). Metal-coordinated supramolecular polymers from the minimalistic hybrid peptide foldamers. Angew. Chem. Int. Ed..

[20] Kieffer M., Garcia A.M., Haynes C.J.E., Kralj S., Iglesias D., Nitschke J.R., Marchesan S. (2019). Embedding and positioning of two Fe^II^_4_L_4_ cages in supramolecular tripeptide gels for selective chemical segregation. Angew. Chem. Int. Ed..

[21] Falcone N., Kraatz H.-B. (2018). Supramolecular assembly of peptide and metallopeptide gelators and their stimuli-responsive properties in biomedical applications. Chem. Eur. J..

[22] McEwen H., Du E.Y., Mata J.P., Thordarson P., Martin A.D. (2017). Tuning hydrogels through metal-based gelation triggers. J. Mater. Chem. B.

[23] Metrano A.J., Chinn A.J., Shugrue C.R., Stone E.A., Kim B., Miller S.J. (2020). Asymmetric catalysis mediated by synthetic peptides, version 2.0: Expansion of scope and mechanisms. Chem. Rev..

[24] Han J., Gong H., Ren X., Yan X. (2021). Supramolecular nanozymes based on peptide self-assembly for biomimetic catalysis. Nano Today.

[25] Arad E., Jelinek R. (2022). Catalytic amyloids. Trends Chem..

[26] Carvalho S., Peralta Reis D.Q., Pereira S.V., Kalafatovic D., Pina A.S. (2022). Catalytic peptides: The challenge between simplicity and functionality. Isr. J. Chem..

[27] Chatterjee A., Reja A., Pal S., Das D. (2022). Systems chemistry of peptide-assemblies for biochemical transformations. Chem. Soc. Rev..

[28] Firipis K., Nisbet D.R., Franks S.J., Kapsa R.M.I., Pirogova E., Williams R.J., Quigley A. (2021). Enhancing peptide biomaterials for biofabrication. Polymers.

[29] Li T., Lu X.M., Zhang M.R., Hu K., Li Z. (2022). Peptide-based nanomaterials: Self-assembly, properties and applications. Bioactive Mater..

[30] Lee S., Trinh T.H.T., Yoo M., Shin J., Lee H., Kim J., Hwang E., Lim Y.B., Ryou C. (2019). Self-assembling peptides and their application in the treatment of diseases. Int. J. Mol. Sci..

[31] La Manna S., Di Natale C., Onesto V., Marasco D. (2021). Self-assembling peptides: From design to biomedical applications. Int. J. Mol. Sci..

[32] Yu Y., Mok B.Y.L., Loh X.J., Tan Y.N. (2016). Rational design of biomolecular templates for synthesizing multifunctional noble metal nanoclusters toward personalized theranostic applications. Adv. Health Mater..

[33] Wang Q., Jiang N., Fu B., Huang F., Liu J. (2019). Self-assembling peptide-based nanodrug delivery systems. Biomater. Sci..

[34] Zhang Z., Ai S., Yang Z., Li X. (2021). Peptide-based supramolecular hydrogels for local drug delivery. Adv. Drug Deliv. Rev..

[35] Diaferia C., Rosa E., Accardo A., Morelli G. (2022). Peptide-based hydrogels as delivery systems for doxorubicin. J. Pept. Sci..

[36] Contini A., Erba E., Bondavalli V., Barbiroli A., Gelmi M.L., Romanelli A. (2021). Morpholino-based peptide oligomers: Synthesis and DNA binding properties. Biochem. Biophys. Res. Commun..

[37] Zeng X.Z., An H.W., Wang H. (2021). Chemical reactions trigger peptide self-assembly in vivo for tumor therapy. ChemMedChem.

[38] Conibear A.C., Schmid A., Kamalov M., Becker C.F.W., Bello C. (2020). Recent advances in peptide-based approaches for cancer treatment. Curr. Med. Chem..

[39] De Rosa L., Di Stasi R., D’Andrea L.D. (2018). Pro-angiogenic peptides in biomedicine. Arch. Biochem. Biophys..

[40] Diaferia C., Gianolio E., Accardo A. (2019). Peptide-based building blocks as structural elements for supramolecular Gd-containing MRI contrast agents. J. Pept. Sci..

[41] Gallo E., Rosa E., Diaferia C., Rossi F., Tesauro D., Accardo A. (2020). Systematic overview of soft materials as a novel frontier for mri contrast agents. RSC Adv..

[42] Li L.L., Qiao Z.Y., Wang L., Wang H. (2019). Programmable construction of peptide-based materials in living subjects: From modular design and morphological control to theranostics. Adv. Mater..

[43] Rai A., Ferrão R., Palma P., Patricio T., Parreira P., Anes E., Tonda-Turo C., Martins M.C.L., Alves N., Ferreira L. (2022). Antimicrobial peptide-based materials: Opportunities and challenges. J. Mater. Chem. B.

[44] Binaymotlagh R., Chronopoulou L., Haghighi F.H., Fratoddi I., Palocci C. (2022). Peptide-based hydrogels: New materials for biosensing and biomedical applications. Materials.

[45] Adedeji Olulana A.F., Soler M.A., Lotteri M., Vondracek H., Casalis L., Marasco D., Castronovo M., Fortuna S. (2021). Computational evolution of beta-2-microglobulin binding peptides for nanopatterned surface sensors. Int. J. Mol. Sci..

[46] Zhang L., Huang Y., Lindstrom A.R., Lin T.Y., Lam K.S., Li Y. (2019). Peptide-based materials for cancer immunotherapy. Theranostics.

[47] O’Neill C.L., Shrimali P.C., Clapacs Z.P., Files M.A., Rudra J.S. (2021). Peptide-based supramolecular vaccine systems. Acta Biomater..

[48] Rajeev N., AL S., Chandran A., Sadanandan S. (2022). Recent advances in peptides-based stimuli-responsive materials for biomedical and therapeutic applications: A review. Mol. Pharm..

[49] Martin A.D., Thordarson P. (2020). Beyond Fmoc: A review of aromatic peptide capping groups. J. Mater. Chem. B.

[50] Tao K., Levin A., Adler-Abramovich L., Gazit E. (2016). Fmoc-modified amino acids and short peptides: Simple bio-inspired building blocks for the fabrication of functional materials. Chem. Soc. Rev..

[51] Sharma P., Kaur H., Roy S. (2019). Inducing differential self-assembling behavior in ultrashort peptide hydrogelators using simple metal salts. Biomacromolecules.

[52] Ji W., Yuan C., Zilberzwige-Tal S., Xing R., Chakraborty P., Tao K., Gilead S., Yan X., Gazit E. (2019). Metal-ion modulated structural transformation of amyloid-like dipeptide supramolecular self-assembly. ACS Nano.

[53] Skopinska-Wisniewska J., De la Flor S., Kozlowska J. (2021). From supramolecular hydrogels to multifunctional carriers for biologically active substances. Int. J. Mol. Sci..

[54] Pal V.K., Roy S. (2022). Cooperative metal ion coordination to the short self-assembling peptide promotes hydrogelation and cellular proliferation. Macromol. Biosci..

[55] Dong L., Chen H., Liu T., Zhu J., Yu M., Yuan Q. (2021). Poly(L-cysteine) peptide amphiphile derivatives containing disulfide bonds: Synthesis, self-assembly-induced β-sheet nanostructures, pH/reduction dual response, and drug release. Biomacromolecules.

[56] Bauer T.A., Eckrich J., Wiesmann N., Kuczelinis F., Sun W., Zeng X., Weber B., Wu S., Bings N.H., Strieth S. (2021). Photocleavable core cross-linked polymeric micelles of polypept(o)ides and ruthenium(II) complexes. J. Mater. Chem. B.

[57] Wu C., Liu J., Zhai Z., Yang L., Tang X., Zhao L., Xu K., Zhong W. (2020). Double-crosslinked nanocomposite hydrogels for temporal control of drug dosing in combination therapy. Acta Biomater..

[58] Zhai Z., Xu K., Mei L., Wu C., Liu J., Liu Z., Wan L., Zhong W. (2019). Co-assembled supramolecular hydrogels of cell adhesive peptide and alginate for rapid hemostasis and efficacious wound healing. Soft Matter.

[59] Zhang Y.-L., Chang R., Duan H.-Z., Chen Y.-X. (2020). Metal ion and light sequentially induced sol–gel–sol transition of a responsive peptide-hydrogel. Soft Matter.

[60] Marin D., Marchesan S. (2022). Self-assembled peptide nanostructures for ECM biomimicry. Nanomaterials.

[61] Zeng L., Song M., Gu J., Xu Z., Xue B., Li Y., Cao Y. (2019). A highly stretchable, tough, fast self-healing hydrogel based on peptide⁻metal ion coordination. Biomimetics.

[62] Tunn I., Harrington M.J., Blank K.G. (2019). Bioinspired histidine⁻Zn^2+^ coordination for tuning the mechanical properties of self-healing coiled coil cross-linked hydrogels. Biomimetics.

[63] Patel M., Lee H.J., Son S., Kim H., Kim J., Jeong B. (2020). Iron ion-releasing polypeptide thermogel for neuronal differentiation of mesenchymal stem cells. Biomacromolecules.

[64] Paladini F., Meikle S.T., Cooper I.R., Lacey J., Perugini V., Santin M. (2013). Silver-doped self-assembling di-phenylalanine hydrogels as wound dressing biomaterials. J. Mater. Sci..

[65] Colovic M.B., Lackovic M., Lalatovic J., Mougharbel A.S., Kortz U., Krstic D.Z. (2020). Polyoxometalates in biomedicine: Update and overview. Curr. Med. Chem..

[66] Li X., Zheng T., Liu X., Du Z., Xie X., Li B., Wu L., Li W. (2019). Coassembly of short peptide and polyoxometalate into complex coacervate adapted for ph and metal ion-triggered underwater adhesion. Langmuir.

[67] Colomina-Alfaro L., Marchesan S., Stamboulis A., Bandiera A. (2022). Smart tools for antimicrobial peptides expression and application: The elastic perspective. Biotechnol. Bioeng..

[68] Bellotto O., Semeraro S., Bandiera A., Tramer F., Pavan N., Marchesan S. (2022). Polymer conjugates of antimicrobial peptides (AMPs) with D-amino acids (D-aa): State of the art and future opportunities. Pharmaceutics.

[69] Garcia A.M., Lavendomme R., Kralj S., Kurbasic M., Bellotto O., Cringoli M.C., Semeraro S., Bandiera A., De Zorzi R., Marchesan S. (2020). Self-assembly of an amino acid derivative into an antimicrobial hydrogel biomaterial. Chem. Eur. J..

[70] Nowak M.G., Skwarecki A.S., Milewska M.J. (2021). Amino acid based antimicrobial agents—Synthesis and properties. ChemMedChem.

[71] Qian Y., Altamimi A., Yates S.A., Sarkar S., Cochran M., Zhou M., Levi-Polyachenko N., Matson J.B. (2020). H_2_S-releasing amphiphilic dipeptide hydrogels are potent *S. aureus* biofilm disruptors. Biomater. Sci..

[72] Saadouli I., Zendah El Euch I., Trabelsi E., Mosbah A., Redissi A., Ferjani R., Fhoula I., Cherif A., Sabatier J.-M., Sewald N. (2020). Isolation, characterization and chemical synthesis of large spectrum antimicrobial cyclic dipeptide (L-leu-L-pro) from *Streptomyces misionensis* V16R3Y1 bacteria extracts. A novel ^1^H NMR metabolomic approach. Antibiotics.

[73] Yu X., Li L., Sun S., Chang A., Dai X., Li H., Wang Y., Zhu H. (2021). A cyclic dipeptide from marine fungus *Penicillium chrysogenum* DXY-1 exhibits anti-quorum sensing activity. ACS Omega.

[74] Sharma K., Aaghaz S., Shenmar K., Jain R. (2018). Short antimicrobial peptides. Recent Pat. Anti-Infect. Drug Discov..

[75] Rosetti B., Scarel E., Colomina-Alfaro L., Adorinni S., Pierri G., Bellotto O., Mamprin K., Polentarutti M., Bandiera A., Tedesco C. (2022). Self-assembly of homo- and hetero-chiral cyclodipeptides into supramolecular polymers towards antimicrobial gels. Polymers.

[76] Carpa R., Remizovschi A., Culda C.A., Butiuc-Keul A.L. (2022). Inherent and composite hydrogels as promising materials to limit antimicrobial resistance. Gels.

[77] Masimen M.A.A., Harun N.A., Maulidiani M., Ismail W.I.W. (2022). Overcoming methicillin-resistance *Staphylococcus aureus* (MRSA) using antimicrobial peptides-silver nanoparticles. Antibiotics.

[78] Skwarczynski M., Bashiri S., Yuan Y., Ziora Z.M., Nabil O., Masuda K., Khongkow M., Rimsueb N., Cabral H., Ruktanonchai U. (2022). Antimicrobial activity enhancers: Towards smart delivery of antimicrobial agents. Antibiotics.

[79] Birkett M., Dover L., Cherian Lukose C., Wasy Zia A., Tambuwala M.M., Serrano-Aroca Á. (2022). Recent advances in metal-based antimicrobial coatings for high-touch surfaces. Int. J. Mol. Sci..

[80] Mishra A., Pradhan D., Halder J., Biswasroy P., Rai V.K., Dubey D., Kar B., Ghosh G., Rath G. (2022). Metal nanoparticles against multi-drug-resistance bacteria. J. Inorg. Biochem..

[81] Song J., Yuan C., Jiao T., Xing R., Yang M., Adams D.J., Yan X. (2020). Multifunctional antimicrobial biometallohydrogels based on amino acid coordinated self-assembly. Small.

[82] D’Souza A., Yoon J.H., Beaman H., Gosavi P., Lengyel-Zhand Z., Sternisha A., Centola G., Marshall L.R., Wehrman M.D., Schultz K.M. (2020). Nine-residue peptide self-assembles in the presence of silver to produce a self-healing, cytocompatible, antimicrobial hydrogel. ACS Appl. Mater. Interfaces.

[83] Schnaider L., Toprakcioglu Z., Ezra A., Liu X., Bychenko D., Levin A., Gazit E., Knowles T.P.J. (2020). Biocompatible hybrid organic/inorganic microhydrogels promote bacterial adherence and eradication in vitro and in vivo. Nano Lett..

[84] Iudin D., Vasilieva M., Knyazeva E., Korzhikov-Vlakh V., Demyanova E., Lavrentieva A., Skorik Y., Korzhikova-Vlakh E. (2022). Hybrid nanoparticles and composite hydrogel systems for delivery of peptide antibiotics. Int. J. Mol. Sci..

[85] Baghdasaryan A., Grillo R., Roy Bhattacharya S., Sharma M., Reginato E., Theraulaz H., Dolamic I., Dadras M., Rudaz S., Varesio E. (2018). Facile synthesis, size-separation, characterization, and antimicrobial properties of thiolated copper clusters. ACS Appl. Nano Mater..

[86] Gatto E., Toniolo C., Venanzi M. (2022). Peptide self-assembled nanostructures: From models to therapeutic peptides. Nanomaterials.

[87] Taş K., Volta B.D., Lindner C., El Bounkari O., Hille K., Tian Y., Puig-Bosch X., Ballmann M., Hornung S., Ortner M. (2022). Designed peptides as nanomolar cross-amyloid inhibitors acting via supramolecular nanofiber co-assembly. Nat. Commun..

[88] Gao K.X., Zhou Z., Yao L., Wang S., Zhang Y., Zou Q., Ma L.X., Wang H.X. (2022). Aspartic acid-assisted size-controllable synthesis of nanoscale spherical covalent organic frameworks with chiral interfaces for inhibiting amyloid-β fibrillation. ACS Appl. Bio Mater..

[89] Soto C., Sigurdsson E.M., Morelli L., Asok Kumar R., Castaño E.M., Frangione B. (1998). Β-sheet breaker peptides inhibit fibrillogenesis in a rat brain model of amyloidosis: Implications for alzheimer’s therapy. Nat. Med..

[90] Garcia A.M., Melchionna M., Bellotto O., Kralj S., Semeraro S., Parisi E., Iglesias D., D’Andrea P., De Zorzi R., Vargiu A.V. (2021). Nanoscale assembly of functional peptides with divergent programming elements. ACS Nano.

[91] Fasae K.D., Abolaji A.O., Faloye T.R., Odunsi A.Y., Oyetayo B.O., Enya J.I., Rotimi J.A., Akinyemi R.O., Whitworth A.J., Aschner M. (2021). Metallobiology and therapeutic chelation of biometals (copper, zinc and iron) in Alzheimer’s disease: Limitations, and current and future perspectives. J. Trace Elem. Med. Biol..

[92] Chaves S., Várnagy K., Santos M.A. (2021). Recent multi-target approaches on the development of anti- Alzheimer’s agents integrating metal chelation activity. Curr. Med. Chem..

[93] Benoit S.L., Maier R.J. (2021). The nickel-chelator dimethylglyoxime inhibits human amyloid beta peptide in vitro aggregation. Sci. Rep..

[94] Wang L., Yin Y.L., Liu X.Z., Shen P., Zheng Y.G., Lan X.R., Lu C.B., Wang J.Z. (2020). Current understanding of metal ions in the pathogenesis of Alzheimer’s disease. Transl. Neurodeg..

[95] Chan T.G., Ruehl C.L., Morse S.V., Simon M., Rakers V., Watts H., Aprile F.A., Choi J.J., Vilar R. (2021). Modulation of amyloid-β aggregation by metal complexes with a dual binding mode and their delivery across the blood-brain barrier using focused ultrasound. Chem. Sci..

[96] Iscen A., Brue C.R., Roberts K.F., Kim J., Schatz G.C., Meade T.J. (2019). Inhibition of amyloid-β aggregation by cobalt(III) Schiff base complexes: A computational and experimental approach. J. Am. Chem. Soc..

[97] La Manna S., Leone M., Iacobucci I., Annuziata A., Di Natale C., Lagreca E., Malfitano A.M., Ruffo F., Merlino A., Monti M. (2022). Glucosyl platinum(II) complexes inhibit aggregation of the C-terminal region of the Aβ peptide. Inorg. Chem..

[98] Manna S., Florio D., Iacobucci I., Napolitano F., Benedictis I., Malfitano A.M., Monti M., Ravera M., Gabano E., Marasco D. (2021). A comparative study of the effects of platinum (II) complexes on β-amyloid aggregation: Potential neurodrug applications. Int. J. Mol. Sci..

[99] Florio D., Malfitano A.M., Di Somma S., Mügge C., Weigand W., Ferraro G., Iacobucci I., Monti M., Morelli G., Merlino A. (2019). Platinum(II) O,S complexes inhibit the aggregation of amyloid model systems. Int. J. Mol. Sci..

[100] Cali M.P., Pereira L.M.B., Teodoro M.D., Sellani T.A., Rodrigues E.G., Carlos R.M. (2021). Comparison of Aβ (1-40, 1-28, 11-22, and 29-40) aggregation processes and inhibition of toxic species generated in early stages of aggregation by a water-soluble ruthenium complex. J. Inorg. Biochem..

[101] Son G., Lee B.I., Chung Y.J., Park C.B. (2018). Light-triggered dissociation of self-assembled β-amyloid aggregates into small, nontoxic fragments by ruthenium (II) complex. Acta Biomater..

[102] Vyas N.A., Singh S.B., Kumbhar A.S., Ranade D.S., Walke G.R., Kulkarni P.P., Jani V., Sonavane U.B., Joshi R.R., Rapole S. (2018). Acetylcholinesterase and Aβ aggregation inhibition by heterometallic ruthenium(II)-platinum(II) polypyridyl complexes. Inorg. Chem..

[103] Peng Y.B., Tao C., Tan C.P., Zhao P. (2021). Inhibition of Aβ peptide aggregation by ruthenium(II) polypyridyl complexes through copper chelation. J. Inorg. Biochem..

[104] Okafor M., Gonzalez P., Ronot P., El Masoudi I., Boos A., Ory S., Chasserot-Golaz S., Gasman S., Raibaut L., Hureau C. (2022). Development of Cu(II)-specific peptide shuttles capable of preventing Cu-amyloid beta toxicity and importing bioavailable Cu into cells. Chem. Sci..

[105] Liu W., Dong X., Sun Y. (2019). D-enantiomeric rthlvffark-NH_2_: A potent multifunctional decapeptide inhibiting Cu^2+^-mediated amyloid β-protein aggregation and remodeling Cu^2+^-mediated amyloid β aggregates. ACS Chem. Neurosci..

[106] Li X., Wang W., Dong X., Sun Y. (2020). Conjugation of rthlvffark to human lysozyme creates a potent multifunctional modulator for Cu^2+^-mediated amyloid β-protein aggregation and cytotoxicity. J. Mater. Chem. B.

[107] Zhang H., Dong X., Sun Y. (2018). Carnosine-lvffark-NH_2_ conjugate: A moderate chelator but potent inhibitor of Cu^2+^-mediated amyloid β-protein aggregation. ACS Chem. Neurosci..

[108] Zhang H., Zhang C., Dong X.Y., Zheng J., Sun Y. (2018). Design of nonapeptide lvffarkhh: A bifunctional agent against Cu^2+^ -mediated amyloid β-protein aggregation and cytotoxicity. J. Mol. Recognit..

[109] Meng J., Zhang H., Dong X., Liu F., Sun Y. (2018). Rthlvffark-NH_2_: A potent and selective modulator on Cu^2+^-mediated amyloid-β protein aggregation and cytotoxicity. J. Inorg. Biochem..

[110] Zhang X., Zhang X., Zhong M., Zhao P., Guo C., Li Y., Xu H., Wang T., Gao H. (2021). A novel Cu(II)-binding peptide identified by phage display inhibits Cu^2+^-mediated Aβ aggregation. Int. J. Mol. Sci..

[111] Zhang X., Zhong M., Zhao P., Zhang X., Li Y., Wang X., Sun J., Lan W., Sun H., Wang Z. (2019). Screening a specific Zn(II)-binding peptide for improving the cognitive decline of alzheimer’s disease in app/ps1 transgenic mice by inhibiting Zn^2+^-mediated amyloid protein aggregation and neurotoxicity. Biomater. Sci..

[112] Shamloo A., Asadbegi M., Khandan V., Amanzadi A. (2018). Designing a new multifunctional peptide for metal chelation and Aβ inhibition. Arch. Biochem. Biophys..

[113] Asadbegi M., Shamloo A. (2019). Identification of a novel multifunctional ligand for simultaneous inhibition of amyloid-beta (Aβ(42)) and chelation of zinc metal ion. ACS Chem. Neurosci..

[114] Zhou B., Sheng X., Xie H., Zhou S., Zhong M., Liu A. (2022). Inhibition of alzheimer’s Aβ(1-42) fibrillogenesis and removal of copper ions by polypeptides modified gold nanoparticles. Chem. Biodivers..

[115] Basu K., Nandi N., Mondal B., Dehsorkhi A., Hamley I.W., Banerjee A. (2017). Peptide-based ambidextrous bifunctional gelator: Applications in oil spill recovery and removal of toxic organic dyes for waste water management. Interface Focus.

[116] Mondal B., Bairagi D., Nandi N., Hansda B., Das K.S., Edwards-Gayle C.J.C., Castelletto V., Hamley I.W., Banerjee A. (2020). Peptide-based gel in environmental remediation: Removal of toxic organic dyes and hazardous Pb^2+^ and Cd^2+^ ions from wastewater and oil spill recovery. Langmuir.

[117] Fortunato A., Mba M. (2021). Metal cation triggered peptide hydrogels and their application in food freshness monitoring and dye adsorption. Gels.

[118] Huang S., Liu X., Hu Q., Wei T., Wang J., Chen H., Wu C. (2020). Temperature-driven metalloprotein-based hybrid hydrogels for selective and reversible removal of cadmium(II) from water. ACS Appl. Mater. Interfaces.

[119] Sfragano P.S., Moro G., Polo F., Palchetti I. (2021). The role of peptides in the design of electrochemical biosensors for clinical diagnostics. Biosensors.

[120] Rozhin P., Charitidis C., Marchesan S. (2021). Self-assembling peptides and carbon nanomaterials join forces for innovative biomedical applications. Molecules.

[121] Gkika K.S., Cullinane D., Keyes T.E. (2022). Metal peptide conjugates in cell and tissue imaging and biosensing. Top. Curr. Chem..

[122] Heaton I., Platt M. (2019). Peptide nanocarriers for detection of heavy metal ions using resistive pulse sensing. Anal. Chem..

[123] Schirone M., Esposito L., D’Onofrio F., Visciano P., Martuscelli M., Mastrocola D., Paparella A. (2022). Biogenic amines in meat and meat products: A review of the science and future perspectives. Foods.

[124] Maiti B., Bhattacharjee S., Bhattacharya S. (2019). Perfluoroarene induces a pentapeptidic hydrotrope into a pH-tolerant hydrogel allowing naked eye sensing of Ca^2+^ ions. Nanoscale.

[125] Wang W., Wang Z., Sun D., Li S., Deng Q., Xin X. (2022). Supramolecular self-assembly of atomically precise silver nanoclusters with chiral peptide for temperature sensing and detection of arginine. Nanomaterials.

[126] Gupta D., Gupta V., Nath D., Miglani C., Mandal D., Pal A. (2022). Stimuli-responsive self-assembly disassembly in peptide amphiphiles to endow block-co-fibers and tunable piezoelectric response. ACS Appl. Mater. Interfaces.

[127] Garifullin R., Guler M.O. (2021). Electroactive peptide-based supramolecular polymers. Mater. Today Bio.

[128] Zhang L., Lu J.R., Waigh T.A. (2021). Electronics of peptide- and protein-based biomaterials. Adv. Coll. Interface Sci..

[129] Piotrowska R., Hesketh T., Wang H., Martin A.R.G., Bowering D., Zhang C., Hu C.T., McPhee S.A., Wang T., Park Y. (2021). Mechanistic insights of evaporation-induced actuation in supramolecular crystals. Nat. Mater..

[130] Newar R., Akhtar N., Antil N., Kumar A., Shukla S., Begum W., Manna K. (2021). Amino Acid-Functionalized Metal-Organic Frameworks for Asymmetric Base–Metal Catalysis. Angew. Chem. Int. Ed..

[131] Shu Y., Fujimoto Y., Taniguchi Y., Miyake K., Uchida Y., Nishiyama N. (2022). Amino-Acid-Functionalized Metal–Organic Frameworks as Excellent Precursors toward Bifunctional Metal-Free Electrocatalysts. ACS Appl. Energy Mater..

[132] Tang H., Yang K., Wang K.-Y., Meng Q., Wu F., Fang Y., Wu X., Li Y., Zhang W.C., Luo Y. (2020). Engineering a homochiral metal–organic framework based on an amino acid for enantioselective separation. Chem. Commun..

